# Multi‐year observations of the high mountain water cycle in the Langtang catchment, Central Himalaya

**DOI:** 10.1002/hyp.14189

**Published:** 2021-05-17

**Authors:** Jakob F. Steiner, Tika R. Gurung, Sharad P. Joshi, Inka Koch, Tuomo Saloranta, Joseph Shea, Arun B. Shrestha, Emmy Stigter, Walter W. Immerzeel

**Affiliations:** ^1^ Department of Physical Geography Utrecht University Utrecht The Netherlands; ^2^ Water and Air International Center for Integrated Mountain Development Kathmandu Nepal; ^3^ Hydrology Department Norwegian Water Resources and Energy Directorate Oslo Norway; ^4^ Geography Program University of Northern British Columbia Prince George British Columbia Canada

## Abstract

The Langtang catchment is a high mountain, third order catchment in the Gandaki basin in the Central Himalaya (28.2°N, 85.5°E), that eventually drains into the Ganges. The catchment spans an elevation range from 1400 to 7234 m a.s.l. and approximately one quarter of the area is glacierized. Numerous research projects have been conducted in the valley during the last four decades, with a strong focus on the cryospheric components of the catchment water balance. Since 2012 multiple weather stations and discharge stations provide measurements of atmospheric and hydrologic variables. Full weather stations are used to monitor at an hourly resolution all four radiation components (incoming and outgoing shortwave and longwave radiation; SW_in/out_ and LW_in/out_), air temperature, humidity, wind speed and direction, and precipitation, and cover an elevational range of 3862–5330 m a.s.l. Air temperature and precipitation are monitored along elevation gradients for investigations of the spatial variability of the high mountain meteorology. Dedicated point‐scale observations of snow cover, depth and water equivalent as well as ice loss have been carried out over multiple years and complement the observations of the water cycle. All data presented is openly available in a database and will be updated annually.

## Graphical abstract

Multiple years of meteorological and hydrological observations as well as the cryosphere from a high mountain catchment in the Central Himalaya are available. Data includes multiple full weather stations throughout the catchment, measuring all components of the energy balance as well as precipitation. Discharge is monitored at the outlet of the catchment.

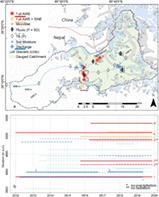

## 1 DATA SET NAME

The Langtang (Central Himalaya) meteorological and streamflow dataset.

## 2 SITE DESCRIPTION

The Langtang catchment, located in the Central Himalaya in Nepal (28.2°N, 85.5°E), covers an area of approximately 585 km^2^ and spans an elevation range from 1400 m a.s.l. at the confluence of the Langtang River with the main Trisuli River to 7234 m a.s.l. at the peak of Langtang Lirung. Approximately 25% of the area is glacierized, with extensive debris cover on glacier termini below 5200 m. The climate is dominated by the monsoon, and characterized by synoptic scale easterly flow in summer (June to September) and westerly flow between October and May, with near surface wind directions modulated by valley circulation. Between 70% and 90% of the total annual precipitation falls during monsoon (Immerzeel et al., 2014) but total precipitation varies from around 2000 mm year^−1^ at 2300 m to less than 1000 mm year^−1^ 14 km further to the east at 3900 m (Immerzeel et al., 2014). The catchment entrance at Syafru Besi (1400 m) is accessible by road, while the rest of the catchment can only be reached on foot or by helicopter. The highest permanent settlement, Kyanjing, is located at 3900 m, close to the main automatic weather station (AWS) and discharge measurement site.

## 3 INSTRUMENTATION

Data from all stations is downloaded manually once or twice a year on site and uploaded to the database annually, and stations are serviced in the process. Data collected during sensor failure or when a station was not upright or otherwise damaged are set to NA. Stations repeatedly failed either due to battery malfunction, damage due to an earthquake or avalanches as well as high flow events. All data is stored in the database as collected and no processing is performed, with the exception of discharge.

### 3.1 Meteorology

**FIGURE 1:**
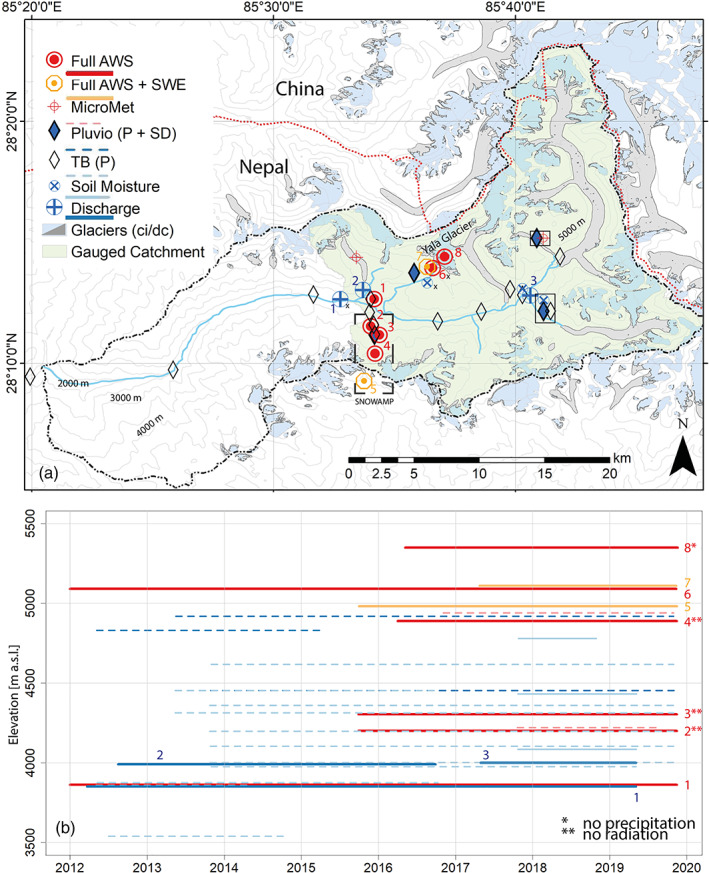
(a) Location of long term measurement sites in Langtang Valley, Nepal. A small x notes the three stations which are shown again in Figure 2. Shaded in light blue is the part of the catchment that is measured by the main discharge station. Glaciers are either clean ice (ci, blue) or debris‐covered (dc, grey). Sensors grouped in a solid black square are all located at the same site as the pluviometer (diamond). (b) Time periods when stations were operational. The maximum time extent shown is until the last field visit in November 2019, while most stations continue to monitor beyond that point in time. AWS, automatic weather station; P, precipitation; SD, snow depth; SWE, snow water equivalent; TB, tipping bucket

Research in the catchment, mainly of the cryosphere, dates back to the 1980s (see Higuchi (1993) for an overview). In 1987 the Government of Nepal through their Department of Hydrology and Meteorology (DHM) established a semi‐automatic meteorological station at Kyanjing (3862 m a.s.l.) and daily measurements of stage height at Langtang Village (3850 m a.s.l; Grabs & Pokhrel, 1993). Automatic stations were installed since 2012, with sub‐daily meteorological and hydrological measurements being collected at numerous sites (Figure 1) through a collaboration between DHM as well as researchers at Utrecht University, ETH Zurich, and the International Centre for Integrated Mountain Development (ICIMOD). The sensor specifications for the main stations are provided in Table 1. Three full AWS are operational at 3862 m a.s.l. (Kyanjing) and 5090 m a.s.l. (Yala Basecamp) and just outside the catchment at Ganja La (SnowAMP, 4962 m a.s.l.; Table 2), measuring all four radiation components (SW_in/out_, LW_in/out_), wind speed and direction, air temperature and relative humidity as well as precipitation and snow depth. Snow depth at all stations in the catchment is stored as distance from sensor to surface and actual snow depth has to be computed by the user. An additional AWS with the same sensors as above, excluding precipitation measurements is operational on Yala Glacier at approximately 5330 m a.s.l. This station is reinstalled as needed due to the wasting ice. All AWS sample data at 10 min interval, which are subsequently aggregated to hourly intervals. Small weather stations (MicroMet; sensor type: Lufft WS500‐UMB, Fellbach, Germany) that measure wind speed (accuracy: ±0.3 m/s or ±3%) and direction (<3° at wind speed >1 m/s), air temperature (±0.2°C) and relative humidity (±2%) are located on debris‐covered ice (Lirung Glacier, 4200 m a.s.l.) and a moraine (Morimoto, 4919 m a.s.l.). In addition a transect of MicroMet stations (sensor type for wind: Lufft WS300‐UMB, Fellbach, Germany; sensor type for temperature and humidity: Campbell Scientific 215, Logan, USA) measuring wind speed and direction (accuracies as above) as well as air temperature (±0.3°C (at 25°C) ±0.4°C (5–40°C)) and relative humidity (±4% at 25°C) was placed on the Yala Plateau, (4800, 5278 and 5504 m a.s.l.) between October 2016 and April 2017 (Stigter et al., 2018), not shown in Figure 1. All MicroMet stations sample at 15 min intervals and data is published in the same format.

**TABLE 1:** Sensor specifications for the AWS and pluviometers

	Radiation	Temperature + humidity	Wind speed/direction	Precipitation	Snow height
Full AWS	Kipp and Zonen *Delft*, *Netherlands* CNR1 (±10%, daily) or CNR4 (<5%, daily)	Rotronic HC2S3 *Bassersdorf*, *Switzerland* (±0.2°C, ±0.8% RH) or CS215 *Logan*, *USA* (±0.3°C, ±4% RH)	CS Young 05108 *Logan*, *USA* (±0.8 m/s)	Ott Pluvio‐2 *Kempten*, *Germany* (±0.05 mm, hourly; ±6 mm/h)	CS SR50AT *Logan*, *USA* (±1 cm)
SNOWAMP^a^	–	CS215 *Logan*, *USA* (±0.3°C, ±4% RH)	CS Young 05108 Alpine *Logan*, *USA* (±0.8 m/s)	Sutron Gage 5600‐0425 *Sterling*, *USA* (±2%)	CS SR50AT *Logan*, *USA* (±1 cm)
Pluviometer	–	Rotronic HC2S3 *Bassersdorf*, *Switzerland* (±0.2°C, ±0.8% RH)	Vector Instruments A100^b^ *Denbighshire*, *UK* (±1%)	Ott Pluvio‐2 *Kempten*, *Germany* (±0.05 mm, hourly; ±6 mm/h)	CS SR50AT *Logan*, *USA* (±1 cm)

*Note*: Sampling interval at all main stations is 10 min, data is available at hourly intervals unless otherwise stated in the metadata. *CS* stands for the manufacturer *Campbell Scientific* (Logan, USA). Location of manufacturer are given in italics after the sensor type. Sensor accuracies are shown in brackets and with the metadata of each station.

^a^ These are the three lower stations along the transect shown in (Figure 1).

^b^ Only at Langshisha pluviometer.

**TABLE 2:** Database locations (doi) for all sensor setups as well as their elevation in the catchment

	Station type	Name	Doi	Elevation [m a.s.l.]
Meteorology	AWS	Kyanjing	https://doi.org/10.26066/RDS.22464	3862
		Yala Basecamp	https://doi.org/10.26066/RDS.26859	5090
		Yala Glacier	https://doi.org/10.26066/RDS.1972507	5330
	Micromet	Yala 1	https://doi.org/10.26066/RDS.1972412	4800
		Yala 2	https://doi.org/10.26066/RDS.1972411	5278
		Yala 3	https://doi.org/10.26066/RDS.1972410	5504
		Lirung	https://doi.org/10.26066/RDS.1972409	4200
		Morimoto	https://doi.org/10.26066/RDS.1972408	4919
	Pluviometer	Yala	https://doi.org/10.26066/RDS.1972420	4919
		Ganja La	https://doi.org/10.26066/RDS.1972415	4361
		Langshisha	https://doi.org/10.26066/RDS.1972414	4452
		Morimoto	https://doi.org/10.26066/RDS.1972413	4831
	Tipping Buckets	Syafrubesi*	https://doi.org/10.26066/RDS.1972430	1406
		Lama Hotel*	https://doi.org/10.26066/RDS.1972421	2370
		Langtang Village*	https://doi.org/10.26066/RDS.1972427	3539
		Kyanjing*	https://doi.org/10.26066/RDS.1972422	3857
		Lirung	https://doi.org/10.26066/RDS.1972426	4141
		Ganja La 1	https://doi.org/10.26066/RDS.1972416	4002
		Ganja La 2	https://doi.org/10.26066/RDS.1972425	4197
		Ganja La 3	https://doi.org/10.26066/RDS.1972424	4361
		Jathang*	https://doi.org/10.26066/RDS.1972423	3875
		Numthang 1* + 2	https://doi.org/10.26066/RDS.1972432 https://doi.org/10.26066/RDS.1972433	3974
		Langshisha Basecamp	https://doi.org/10.26066/RDS.1972429	4104
		Shalbachum	https://doi.org/10.26066/RDS.1972431	4312
		Langshisha Pluviometer	https://doi.org/10.26066/RDS.1972428	4452
		Morimoto Basecamp	https://doi.org/10.26066/RDS.1972434	4617
	Eddy covariance	Lirung	https://doi.org/10.26066/RDS.1972547	4200
		Yala	https://doi.org/10.26066/RDS.1972551	5350
Cryosphere	SNOWAMP	lower	https://doi.org/10.26066/RDS.1972502	4200
		middle	https://doi.org/10.26066/RDS.1972503	4304
		upper	https://doi.org/10.26066/RDS.1972504	4888
		Ganja La (main)	https://doi.org/10.26066/RDS.1972505	4962
	SWE station Yala Plateau		https://doi.org/10.26066/RDS.1972554	5090
	GST loggers		https://doi.org/10.26066/RDS.1972553	4520–5542
	Mass balance Yala Glacier		https://doi.org/10.5904/wgms‐fog‐2020‐08	NA
	UAV data		https://doi.org/10.5281/zenodo.3824264	NA
Hydrology	Discharge	Kyanjing	https://doi.org/10.26066/RDS.1972407	3850
		Lirung	https://doi.org/10.26066/RDS.1972406	3990
		Langshisha (old and new)	https://doi.org/10.26066/RDS.1972404 https://doi.org/10.26066/RDS.1972405	4069 4000
	Soil moisture	Tserko Ri	https://doi.org/10.26066/RDS.1972417	4807
		Langshisha Basecamp	https://doi.org/10.26066/RDS.1972419	4104
		Langshisha Pluviometer	https://doi.org/10.26066/RDS.1972418	4452

*Note*: Tipping Buckets marked with a * were installed in 2012.

For 2 weeks on Lirung Glacier (Steiner et al., 2018; independent station) and multiple weeks on Yala Glacier (Stigter et al., 2018; mounted on the Yala Glacier AWS mentioned above) eddy covariance was measured to determine turbulent exchanges over the glacier surface, using the Campbell Scientific IRGASON sensor (Logan, USA). Data is collected at 10 Hz interval and stored at 5 and 60 min interval for Lirung and Yala respectively. On Lirung Glacier, low frequency radiation (CNR1), air temperature and relative humidity (HC2S3) is also available, sampled and stored in 10 min intervals.

A network of sensors was installed to observe spatiotemporal variability in precipitation. Pluviometers at 4919 m a.s.l. (Morimoto, Table 2) on the northern slope and 4452 m a.s.l. (Langshisha) on the southern slope measured total precipitation, snow height and wind speed, temperature and relative humidity (Table 1), and allowed for an analysis of meteorological conditions during precipitation events. Two additional pluviometers at lower elevations were operational for shorter periods of time at 4361 m a.s.l. (Ganja La) on a southern slope and 4831 m a.s.l. (Yala) on a northern slope. To investigate liquid precipitation gradients in the catchment six unheated tipping buckets (ARG100, Campbell Scientific, Logan, USA; accuracy: ±1% at 0.5 m/h) were placed along the main valley in 2012 (Immerzeel et al., 2014), with additional eight sensors operational since 2013 (Casella, Sycamore, USA; accuracy: ±2% at 1 L/h; Table 2). These sensors store the time step at each individual tip, which then needs to be aggregated by the user to a final time interval and associated precipitation volume. Precipitation data is not corrected for undercatch. However wind speed is recorded alongside pluviometers which allows for simple corrections that are especially relevant during snowfall (Thériault et al., 2012).

### 3.2 Cryosphere

Snow height and snow water equivalent (SWE) are monitored at two locations in the catchment. On the Yala Plateau (5090 m a.s.l.) a gamma ray sensor monitors SWE (Campbell Scientific 725, Logan, USA; accuracy: ±15 mm (0–300 mm), ±15% (300–600 mm); sampling and storage of 24 h averages every 6 h) concurrently with snow height (Campbell Scientific SR50A, Logan, USA; accuracy: ±1 cm; 10 min sampling and 60 min storage interval) and all four radiation components (Kipp and Zonen CNR4, Delft, Netherlands; accuracy: <5% for daily values; 10 min sampling and 60 min storage interval). On the same plateau, to the south of Yala Glacier, a total of 35 near‐surface ground temperature loggers were deployed in 2013 and 2020 between 4520 and 5542 m a.s.l. by ICIMOD and international partners to monitor the local permafrost development (GST, Table 2; Gruber et al., 2017). They include 23 sensors from Geoprecision (Ettlingen, Germany; M‐Log5W; accuracy: ±0.1°C; precision: 0.01°C), 4 from Geotest with the same accuracy (Zollikofen, Switzerland; UTL‐3) as well as 8 iButtons (Whitewater, USA; DS122L; accuracy: ±0.5°C; precision: 0.05°C; discontinued in 2014). All near‐surface ground temperature loggers were logging at a 60 min interval until 2016 and at a 20 min interval since then and are stored at 60 min interval.

Three AWS (referred to as SNOWAMP, Tables 1 and 2; Figure 1) have been operational along a north‐facing slope since 2015 at 4200, 4304 (until November 2018) and 4888 m a.s.l., complementing the full AWS at 4962 m a.s.l. (Saloranta et al., 2016). These sites include direct measurements of SWE at the highest site (Campbell Scientific 725, Logan, USA; accuracy: ±15 mm (0–300 mm), ±15% (300–600 mm)) and snow depth measurements on all other locations (Table 1; Kirkham et al., 2019). These stations sample and store all variables at 60 min intervals, with the exception at the full AWS where wind speed and direction are sampled every 15 min and for SWE 24 h averages are stored every 6 h.

Yala Glacier is one of the few glaciers in High Mountain Asia where mass balance is bi‐annually measured and reported to the World Glacier Monitoring Service (WGMS, Zemp et al., 2020). Over the lower ablation area of two debris‐covered glaciers (Lirung and Langtang), repeat high resolution digital elevation models between 2013 and 2018 are available that allow a quantification of surface height changes and debris‐covered glacier dynamics.

### 3.3 Hydrology

Stage height was measured on the main stem of Langtang River at 3850 m a.s.l. using a radar level sensor (Table 2, Ott RLS, Kempten, Germany; accuracy: ±10 mm; sampling at 10 min and storage at 60 min intervals) from 2012 to 2014 that was subsequently replaced by a pressure level sensor from the same producer (Ott PLS; accuracy: 0.05%). Both setups were drilled into rock and data was collected in a datalogger (Campbell Scientific CR800) approximately 10 m above the river. Using rhodamine and salt dilution techniques, at least twice before and twice after monsoon each year, two separate rating curves were developed for low flow and high flow, as the river channel widens significantly at a stage height of 1.4 m and the rating curve has a lower slope. Over a limited period of time, stage height was monitored at the outlet of Lirung Glacier (2012–2016; sampling ay 10 min and storage at 60 min interval; Ott PLS; Ragettli et al., 2015) and Langshisha Glacier (2013–2017; Ott PLS; sampling at 10 min and storage at 60 min interval), the latter of which had to be shifted 1 km downstream after a high flow event destroyed the station and a new setup was installed (2017–2019; HOBO U20L‐01; Onset, Bourne, USA; accuracy: ±0.1%/1 cm). For all these locations single rating curves were adequate. Both stage height, rating curve parameters and discharge are provided in the database together with the standard error calculated for each stage height based on the uncertainty from the rating curve. The standard error at the main discharge station is highest for low flow (>1 m^3^ s^−1^ for stage height <0.4 m) and lowest for medium flow condition (~0.13 m^3^ s^−1^ at 0.9 m). Periods during which sensor displacement occurred and accurate depths could not be determined with full confidence are removed. Both stage height and computed discharge are available on the database, in combination with all rating curve parameters. To monitor transient subsurface water storage of the catchment, three soil moisture stations at 4104, 4452 and 4807 m a.s.l. (Table 2, HOBO S‐SMC‐M005, Onset, Bourne, USA; accuracy ±0.3%/±0.031 m^3^/m^3^; sampling and storage interval: 5 min) provide measurements from three different depths (0.1 to 0.5 m), respectively, since autumn 2017.

## 4 RESEARCH INSIGHTS

**FIGURE 2:**
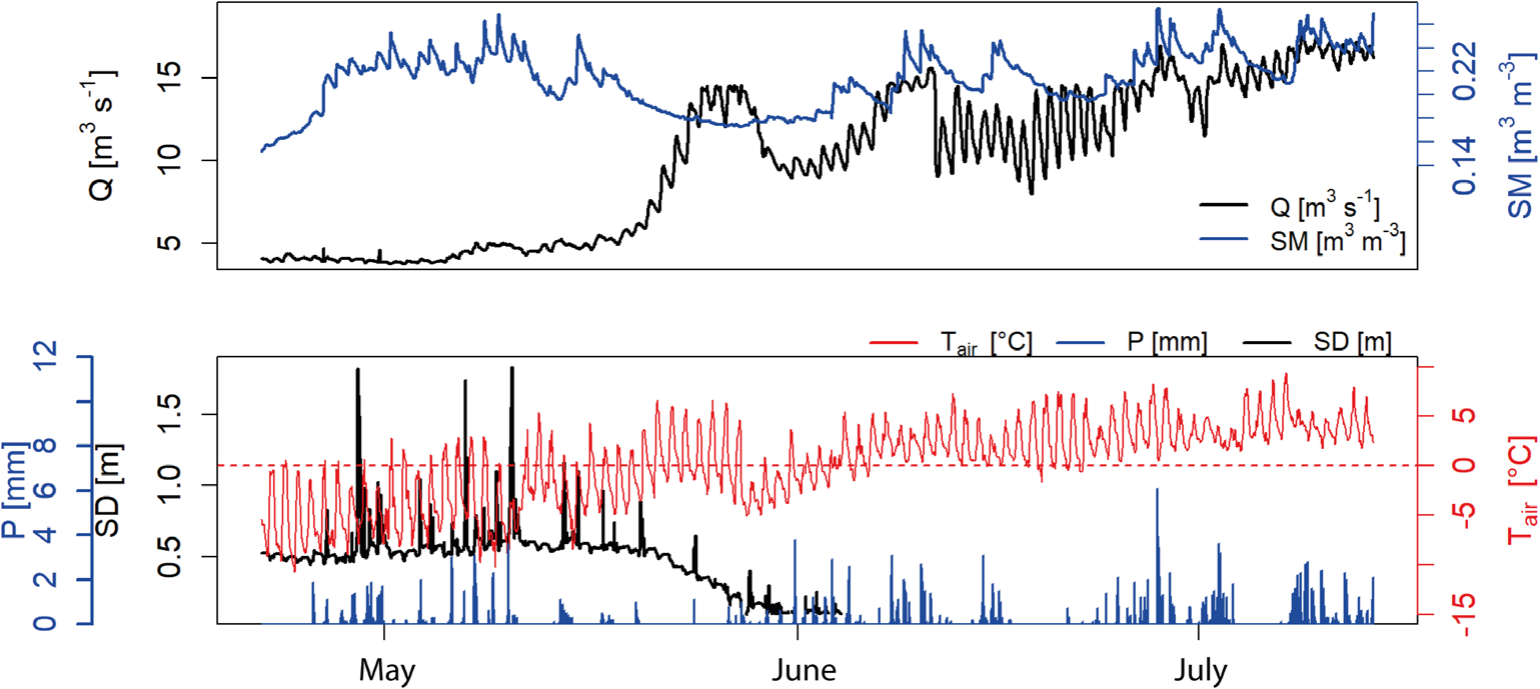
Discharge (Q), snow depth (SD), soil moisture (SM), precipitation (P) and air temperature (T_air_) data from 21 April to 14 July 2018. The dashed red line marks the 0**°**C line. Note that discharge is measured at 3850 m a.s.l., soil moisture at 4807 m a.s.l. and all other variables at 5100 m a.s.l.

Discharge measured at the main gauging station varies between below 5 m^3^ s^−1^ (winter) and 20 m^3^ s^−1^ (monsoon, Figure 2). A steep increase in discharge occurs in May, when snow melt at high altitudes coincides with the first monsoon rains in lower regions, soil thaws and becomes saturated (Figure 2). Variability in soil moisture after saturation is driven by precipitation events that result in high altitude snow fall and almost immediate melt shortly thereafter. By early June, the soil is near‐saturated due to the snow melt, monsoon precipitation begins, and high elevation snow and ice melt is occurring. As a result the discharge rises rapidly, and has marked diurnal peaks. From then onwards, precipitation events measured at 5000 m a.s.l. are clearly and immediately visible in both soil moisture and discharge in the valley (Figure 2).

The streamflow decreases steeply in September as the monsoon withdraws. A distinct diurnal cycle is observed throughout the year, with low/high flows in the late morning/early afternoon in January and early afternoon/midnight in May, respectively.

The availability of multiple measured variables at different elevations, provides the opportunity to evaluate multiple processes at the catchment scale and validate models, especially related to the cryosphere and complex precipitation patterns typical for high mountain regions.

Previous research in the catchment shows a strong elevational gradient of precipitation, which peaks above 1500 mm annually around 3000 m a.s.l. (Collier & Immerzeel, 2015) and rapidly decreases up‐ and down‐valley, with station data showing a drop from 1819 mm at 2370 m a.s.l. to just 867 mm at 3857 m a.s.l. (Immerzeel et al., 2014). At higher elevations the valley floors are generally drier than the southern and northern slopes that receive more orographic precipitation (Collier & Immerzeel, 2015). Temperatures decrease from average 15°C around 1500 m a.s.l. to 0°C at 5000 m a.s.l. Heterogeneous precipitation and temperature patterns drive variability in snow cover (Girona‐Mata et al., 2019) and snow depth (Stigter et al., 2017). Areal snow melt is highly complex due to the extreme relief in the valley, patchy snow cover, and delayed melt output caused by refreezing of melt water within the snow pack (Saloranta et al., 2019). Melt contributions from glacier ice are additionally impacted by a thick layer of debris on the main glacier tongues, which inhibits melt and delays the melt peak.

## 5 DATA

### 5.1 Contributors and ownership of the data

Data collection, processing and quality control was carried out by Tika Gurung, Sharad Joshi (AWS, mass balance and permafrost data), Walter Immerzeel, Inka Koch, Joseph Shea, Jakob Steiner, Emmy Stigter (AWS, all other sensor setups) and Tuomo Saloranta (SnowAMP). Arun Shrestha provided guidance on the database and manuscript development. Jakob Steiner and Walter Immerzeel initiated the Data Note and wrote the manuscript with inputs from all co‐authors. Data are co‐owned by ICIMOD, DHM (all setups), Utrecht University (all setups excl. SNOWAMP) and NVE (SNOWAMP stations). The views and interpretations in this publication are those of the authors and are not necessarily attributable to their organizations.

## Data Availability

Data from the AWS and precipitation measurements as well as all discharge data are available and updated via the regional database of ICIMOD (full URL for all combined data: http://rds.icimod.org/Home/Data?any=Langtang). To access data in the database, an initial registration is necessary, which enables immediate access. Each individual dataset includes a brief description of the station, while metadata describing sensor specifications as well as details on the derivation of discharge data including original stage height measurements is supplied with the data file upon download. Each individual dataset has a separate DOI provided in the respective metadata. Raw data or any additional details on sensor setups and site conditions for all stations may be acquired via the authors. Code to aggregate and quality check data is stored and updated on github for atmospheric (https://github.com/fidelsteiner/AWSprocessing) and discharge data (https://github.com/fidelsteiner/DischargeProcessing).
